# Intraocular pressure and cardiovascular effects of dexmedetomidine premedication and tiletamine-zolazepam for anesthetic induction in dogs

**DOI:** 10.14202/vetworld.2022.2929-2936

**Published:** 2022-12-27

**Authors:** Pradipa Kusolphat, Tanawan Soimala, Jutapoln Sunghan

**Affiliations:** Faculty of Veterinary Science, Prince of Songkla University, 90110 Songkhla, Thailand

**Keywords:** cardiovascular, dexmedetomidine, intraocular pressure, tiletamine-zolazepam

## Abstract

**Background and Aim:**

The effect of anesthetic drugs on intraocular pressure (IOP) is an important concern in ophthalmic surgery. The impact of dexmedetomidine (DEX) combined with tiletamine-zolazepam on IOP is scarcely studied. This study aimed to evaluate IOP and cardiovascular effects in dogs after premedication with 5 μg/kg (DEX5) or 10 μg/kg (DEX10) of intramuscular DEX followed by intravenous tiletamine-zolazepam administration for induction of anesthesia in healthy dogs.

**Materials and Methods:**

Eighteen dogs, American Society of Anesthesiologists I or II, without ocular abnormality were investigated. All dogs were randomly divided into the DEX5 (n = 9) and DEX10 groups (n = 9). The IOP, heart rate (HR), systolic blood pressure (SBP), oxygen saturation, and sedation scale were measured before premedication (baseline), after premedication at 5, 10, 15, and 20 min, after tiletamine-zolazepam administration, after endotracheal intubation, and post-operative.

**Results:**

There were no significant differences between the groups at any time point. The DEX5 and DEX10 groups had significantly decreased HR values at 10 min compared with baseline. The IOP at 20 min was significantly lower compared to the baseline in the DEX10 group. Moreover, the DEX10 group showed increased IOP, HR, SBP, and sedation scale values after induction and intubation compared with 20 min, but these values did not differ significantly from baseline. All parameters of both groups did not change significantly between post-operative and baseline.

**Conclusion:**

Intramuscular DEX (10 μg/kg) is an appropriate premedication in ophthalmic examination or surgical procedures. Moreover, it could be combined with tiletamine-zolazepam for generalized anesthesia in dogs with an ophthalmic problem, as it had no clinically significant effects on IOP or cardiovascular values.

## Introduction

Intraocular pressure (IOP) indicates the ocular perfusion pressure during ophthalmic surgery. An elevated IOP decreases ocular blood flow [1], blocking the transport of neutrophils from the brain, and causing ischemia and optic nerve edema [2, 3]. A suitable anesthetic protocol for ophthalmic surgery should describe maintaining IOP within the normal range or minimizing changes throughout the perioperative period [4, 5]. Moreover, some anesthetic drugs may induce permanent ocular damage, particularly in patients with near-perforating corneal lesions or glaucoma [6].

Dexmedetomidine (DEX) is widely used in veterinary medicine to produce sedation and analgesia for minor surgical and diagnostic procedures, providing premedication before generalized anesthesia [7]. Dexmedetomidine is an alpha-2 adrenergic agonist that lowers IOP and is commonly used in combination with ketamine [8]. Healthy dogs receiving sedation with DEX have a significantly lower heart rate (HR) and IOP, higher systolic blood pressure (SBP) [9], and decreased cardiac output, along with increased systemic vascular resistance [10].

Similarly, tiletamine-zolazepam is widely used in veterinary medicine to induce sedation [11], induction, and generalized anesthesia [12, 13] for diagnostic and surgical procedures in dogs [14, 15]. Tiletamine is a dissociative agent pharmacodynamically similar to ketamine but more potent and with a longer duration of action than ketamine [16, 17]. Moreover, an increase in IOP of approximately 40%–45% is observed at 5 min compared with baseline after ketamine injection [18]. Hofmeister *et al*. [19] have shown that the administration of ketamine alone (5 or 10 mg/kg) and a ketamine-diazepam combination (ketamine 10 mg/kg with diazepam 0.5 mg/kg) can cause a significant increase in IOP in dogs. However, intravenous tiletamine-zolazepam administration has no significant effect on IOP in dogs [16].

A previous study [4] on the effects of DEX and tiletamine-zolazepam on IOP and cardiovascular parameters in dogs were documented. Therefore, this study aimed to evaluate the IOP and cardiovascular effects of different doses of intramuscular DEX following intravenous tiletamine-zolazepam for anesthetic induction in healthy dogs.

## Materials and Methods

### Ethical approval and informed consent

The protocol was approved by the Institutional Animal Care and Use Committee, Prince of Songkla University (Approval No. 2564-05-028). The owners signed a consent form before their animals were included in the study.

### Study period and location

The study was conducted from July 2021 to September 2021 at the Small Animal Hospital, Faculty of Veterinary Science, Prince of Songkla University, Songkhla, Thailand.

### Animals

This study used 18 dogs, ranging from 1 to 5 years of age and any breeds, presented to the Small Animal Hospital, Prince of Songkla University, Thailand, for ovariohysterectomy or castration. All dogs were healthy and American Society of Anesthesiologists (ASA) categories I and II. Moreover, a physical examination was performed, and pre-operative complete blood count and serum chemistry profile (alanine aminotransferase, alkaline phosphatase, blood urea nitrogen, and creatinine) were within normal reference ranges. A thorough ophthalmic examination consisting of Schirmer’s tear test, slit-lamp biomicroscopy, indirect ophthalmoscopy, and IOP measurement by a blinded ophthalmologist was used to check for ocular abnormalities. Exclusion criteria were ASA status of more than II, abnormal ophthalmic examination, dogs that were difficult to restrain for ophthalmic examination, brachycephalic breeds, or aggressive behavior. Feeding was withdrawn 8 h before the experiment, but the animals had free access to water.

### Experimental design

#### Anesthetic protocol

All dogs were allowed to rest for 30 min before any medication. Dogs were randomly assigned to two treatment groups, premedication with DEX intramuscularly (Dexdomitor, Orion Pharma, Finland; 0.5 mg/mL) at 5 μg/kg (DEX5 group) or 10 μg/kg (DEX10 group). After the onset of sedation, an intravenous catheter was placed in the cephalic vein, and generalized anesthesia was induced with tiletamine-zolazepam (3 mg/kg, intravenously, Zoletil^®^, Virbac, France; Zoletil powder diluted with 5 mL of sterile water for injection; 50 mg/mL tiletamine and 50 mg/mL zolazepam) until loss of jaw tone. Then, endotracheal intubation was performed, and anesthesia was maintained with inhaled isoflurane (AERRANE isoflurane USP, Baxter, USA) in 100% oxygenation using a rebreathing circuit. Lactate Ringer’s solution (Lactated Ringer’s injection, GHP, Thailand) was intravenously administered at 10 mL/kg/h. All dogs were given analgesic drugs intravenously before surgery.

After the completion of the surgical procedure, all dogs were injected intravenously with 0.1 mg/kg of atipamezole hydrochloride (Antisedan, Orion Pharma, Finland; 5 mg/mL).

#### Experimental measurement

Measurement and recording of IOP, HR, and SBP by a Doppler ultrasonic sphygmomanometer (Vmed Vet-Dop2™, Vmed Technology, Washington, United States), oxygen saturation (SpO_2_) (PM-60 VET, Mindray, China), and sedation scale were made on all dogs by a veterinarian before receiving premedication (baseline) at 5, 10, 15, and 20 min after premedication, immediately after tiletamine-zolazepam administration, after endotracheal intubation, and at 72 h post-operative.

The IOP value was measured using tonometry (Icare^®^ TONOVET (TV01), Icare Finland Oy, Helsinki, Finland). Reading was obtained from the axial cornea with the probe’s point of contact perpendicular and held approximately 4 mm from the corneal surface. The IOP values of both eyes were measured, and IOP data were recorded as the average of three measurements. Only the IOP value measurement was within an acceptable standard deviation (<2.5% variance), as indicated on the instrument display. IOPs were measured during sternal recumbency. The head remained above the level of the heart and caution was taken not to compress the jugular vein.

The sedation scale was adapted from Grint *et al*. [20] and was a simple descriptive score, with higher sedation scores indicating a greater level of sedation. Seven items were evaluated, namely, spontaneous posture, palpebral reflex, eye position, jaw and tongue relaxation, response to noise, resistance when laid into lateral recumbency, and general appearance. The total sedation scale was assessed as 0–2 = little/no sedation, 4–11 = moderate sedation, and 13+ = heavy sedation [21].

### Statistical analysis

Data were described as the mean with a standard error of the mean. The Shapiro–Wilk test was performed to confirm the normal distribution. The IOP, HR, SBP, and SpO_2_ values were compared between two groups using independent t-tests, whereas the Mann–Whitney U-test was used to evaluate the sedation scale. All data recorded at each time point were compared with baseline and 20 min (pre-induction), using one-way analysis of variance, followed by Dunnett’s tests to identify differences within each group. Statistical analyses were performed using Prism version 9.2.0 (GraphPad Software, Inc., CA, USA); the significance level was set at p < 0.05.

## Results

In total, 18 dogs (nine dogs in each group) were enrolled in this study, 10 males and eight females. The mean body weight was 16.34 ± 1.02 kg (range: 6.4–22.2 kg), and the mean age was 2.80 ± 0.44 years. There were mixed breeds (n = 12), Thai Bangkaew (n = 4), Golden retriever (n = 1), and Beagle (n = 1). The IOP values from the left and the right eye were averaged for each dog at each time point before data analysis as there was no significant difference between IOP in the left and right eye in DEX5 and DEX10 groups (p *=* 0.899 and p *=* 0.211, respectively).

Baseline IOP and cardiovascular variables are presented as the mean and standard error of the mean (Supplementary Table-1). There was no significant difference between the DEX5 and the DEX10 groups in baseline parameters, including IOP (p = 0.456), HR (p = 0.181), SBP (p = 0.941), SpO_2_ (p = 0.814), and sedation scale (p = 0.168). Moreover, no statistically significant differences in IOP, HR, SBP, SpO_2_, and sedation scale were observed between DEX5 and DEX10 at any time point.

**Supplementary Table-1:** Mean ± standard error of the mean of various parameters after administration of 5 and 10 μg/kg dexmedetomidine at each time point (each group, n = 9).

Parameter measurement	Group	Baseline	After dexmedetomidine administration				ATZ	AET	POD

			5 min	10 min	15 min	20 min			
IOP (mmHg)	DEX5	14.83 ± 1.03	14.11 ± 0.80	12.50 ± 0.62	12.06 ± 0.55	12.06 ± 0.47	16.61 ± 1.62	16.22 ± 1.37	16.56 ± 1.15
	DEX10	16.22 ± 1.50	13.17 ± 0.95	12.67 ± 0.90	11.28 ± 0.49	10.72 ± 0.74^a^	15.17 ± 1.63	16.93 ± 1.50^d^	21.00 ± 6.45^e^
HR (beats/min)	DEX5	131.30 ± 10.34	83.33 ± 8.23	72.44 ± 8.85^a^	64.67 ± 6.85^b^	57.67 ± 6.13^c^	124.40 ± 9.00^f^	122.20 ± 6.75^f^	125.30 ± 9.13^f^
	DEX10	113.40 ± 7.55	77.56 ± 6.33	59.33 ± 6.60^b^	55.33 ± 5.16^b^	51.11 ± 5.66^c^	101.10 ± 9.33^d^	109.20 ± 5.37^f^	113.10 ± 7.39^f^
SBP (mmHg)	DEX5	139.56 ± 8.52	135.44 ± 10.41	133.56 ± 8.41	121.78 ± 7.11	123.78 ± 9.34	154.89 ± 10.85	158.44 ± 9.24^d^	150.33 ± 9.68^d^
	DEX10	140.56 ± 10.11	124.11 ± 7.23	127.33 ± 6.90	120.44 ± 6.49	115.33 ± 4.90	154.67 ± 5.43^e^	147.89 ± 6.58	139.89 ± 8.30
SpO_2_ (%)	DEX5	97.56 ± 0.58	95.44 ± 0.78	96.00 ± 0.97	96.33 ± 0.73	96.89 ± 1.03	93.33 ± 1.93	94.67 ± 1.25	97.00 ± 0.50
	DEX10	97.33 ± 0.73	95.44 ± 1.13	93.89 ± 0.86	94.44 ± 0.96	94.44 ± 1.11	90.78 ± 1.51^a^	93.67 ± 1.00	96.33 ± 0.41

IOP=Intraocular pressure, HR=Heart rate, SBP=Systolic blood pressure, SpO_2_=Oxygen saturation, ATZ=After tiletamine-zolazepam, AET=After endotracheal intubation, POD=at 72 h post-operative, ^a,b,c^significantly different within the group from the baseline value;

a p < 0.05;

b p < 0.01; and

c p < 0.001. d,e,fSignificantly different within the group 20 min after premedication;

d p < 0.05;

e p < 0.01; and

f p < 0.001

**Figure-1:**
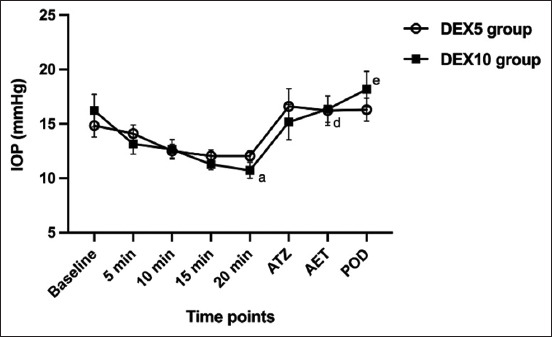
Intraocular pressure measured mean ± standard error of the mean at each time point; before dexmedetomidine administration (baseline), after dexmedetomidine administration at 5, 10, 15, and 20 min, after tiletamine-zolazepam administration, after endotracheal intubation, and at 72 h post-operative. ^a^Significant difference within the group from baseline value; ^a^p < 0.05, ^d,e^significant difference within the group from 20 min after premedication; ^d^p < 0.05 and ^e^p < 0.01.

Following premedication with DEX in both DEX5 and DEX10 groups, IOP was below baseline at 5, 10, 15, and 20 min (Figure-1). Significant decreases in IOP were detected in the DEX10 group at 20 min (p = 0.042) compared to baseline (Figure-1). After tiletamine-zolazepam administration and endotracheal intubation, the IOP increased immediately in both groups; the level in the DEX10 group was significantly higher after endotracheal intubation compared with 20 min after premedication (p = 0.012); however, there was no significant difference from the baseline value (p > 0.999).

**Figure-2:**
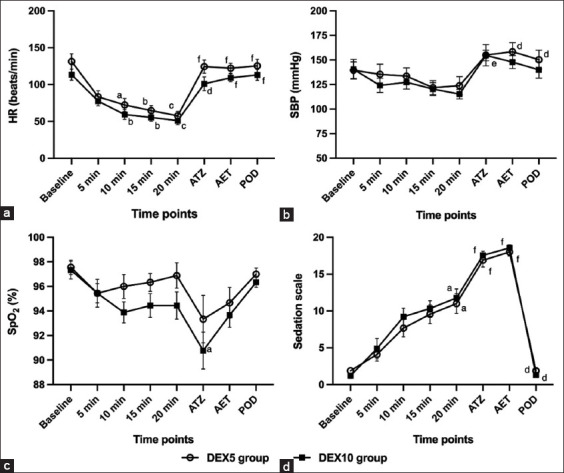
Changes in cardiovascular parameters described as (a) heart rate, (b) systolic blood pressure, (c) oxygen saturation, and (d) sedation scale. Values were determined before dexmedetomidine administration (baseline), after dexmedetomidine administration at 5, 10, 15, and 20 min, after tiletamine-zolazepam administration, after endotracheal intubation, and at 72 h post-operative. ^a,b,c^Significant difference within the group from baseline value; ^a^p < 0.05; ^b^p < 0.01; and ^c^p < 0.001, ^d,e,f^significant difference within the group from 20 min after premedication; ^d^p < 0.05; ^e^p < 0.01; and ^f^p < 0.001. Results are mean ± standard error of the mean.

The HR was significantly lower than baseline after premedication in DEX5 and DEX10 groups at 10 min (p = 0.025 and p = 0.001, respectively), 15 min (p = 0.001 and p = 0.002, respectively), and 20 min (p<0.001) (Figure-2a). After tiletamine-zolazepam administration, HR was significantly increased in the DEX5 group (p < 0.001) and the DEX10 group (p = 0.018) and was still significantly higher after endotracheal intubation in both groups (p < 0.001) compared with 20 min; however, it was not differ significantly different from baseline (p > 0.999).

The SBP was significantly increased after injection of tiletamine-zolazepam in the DEX10 group (p = 0.009) and after endotracheal tube intubation in the DEX5 group (p = 0.005) compared to 20 min after premedication (Figure-2b). The percentage of SpO_2_ showed a statistically significant decrease after tiletamine-zolazepam administration in the Dex10 group (p = 0.025) compared to baseline (Figure-2c).

The sedation scale was significantly increased within DEX5 and Dex10 groups at 20 min (p = 0.010), after tiletamine-zolazepam administration (p < 0.001), and after endotracheal intubation (p < 0.001) compared with baseline (Figure-2d).

Among post-operative measurements, compared with 20 min after premedication, IOP was significantly higher in the DEX10 (p = 0.003) group. Both DEX5 and DEX10 groups showed a significant increase in HR (p < 0.001) and a decrease in sedation scale (p = 0.012 and p = 0.021, respectively), whereas these values were not differ significantly from baseline (p > 0.999).

## Discussion

Successful ophthalmic surgery may depend on sufficient control of IOP before, during, and after the surgery [4]. In dogs, the normal IOP ranges from 10 to 26 mmHg [4] and a low-normal IOP is usually desirable in intraocular surgery [22]. Alpha-2 adrenergic agonists are potent sedative and analgesic drugs in small animal anesthesia [23]. Meanwhile, DEX is widely used in both medicine and veterinary practice, having a dose-related sedative effect with peak action in 15–20 min after intramuscular administration. Studies in human medicine [24] and veterinary medicine show that DEX administration decreases the IOP [4]. Chandra *et al*. [25] studied the effects of intravenous DEX premedication on changes in IOP. They concluded that DEX at 0.4 μg/kg caused a significant decrease in IOP about 5 min after administration. Artigas *et al*. [26] reported that intravenously given DEX (5 μg/kg) reduced the IOP 20 min after drug administration in 42 dogs, which was consistent with our findings. In our study, an intramuscular DEX dose of 10 μg/kg caused a significant decrease in IOP at 20 min.

A previous study reported that three major factors affecting IOP are the volume of aqueous humor production, intraocular (choroidal) blood volume, vitreous humor volume, and extraocular muscle tone [27]. Reitsamer *et al*. [28] explained that the alpha-2 adrenergic agonist decreases ciliary blood flow, ciliary Po_2_, aqueous production, episcleral venous pressure, and IOP. The major mechanism includes stimulating alpha-2 adrenergic receptors, leading to vasoconstriction of the vascular supply of the ciliary process and the episcleral vessels; moreover, a reduction in ciliary blood flow has been associated with a decrease in aqueous production [29, 30].

The tear film is crucial and provides initial protection on the ocular surfaces, including eyelid conjunctiva and cornea. Many studies have shown that tranquilizers, sedatives, opioids, and general anesthetic drugs affect tear production [4]. For example, Kilic and Sarierler [31] reported that tear production was significantly depressed after the dogs were sedated with propionyl promazine-fentanyl, diazepam-fentanyl, propofol, and thiopental. In addition, Dartt [32] found that the combinations of diazepam with butorphanol, acepromazine with oxymorphone, and xylazine with butorphanol decreased tear production, which may induce corneal ulcers in dogs after prolonged anesthesia [33], and moisturizing lubricant eye drops have been recommended during anesthesia to prevent corneal discomfort [34]. However, the evaluation of decreasing tear production was beyond the scope of this study.

Bradycardia is commonly seen after alpha-2 adrenergic agonist administration due to central sympatholytic action [23], which agrees with our finding that both 5 and 10 mg/kg of intramuscular DEX resulted in a significant decrease in HR for the first 10 min after drug administration, but with no significant change in SBP. This result is consistent with other studies that evaluated the hemodynamic effect in dogs after DEX administration. The hemodynamic effect has been explained as a biphasic blood pressure response to decreased HR and cardiac index, as well as increased systemic vascular resistance index and central venous pressure [23].

The combination of an alpha-2 adrenergic agonist with ketamine or tiletamine-zolazepam increased their efficacy and reduced the adverse effects [35–37]. Sympathetic stimulation of tiletamine showed increased HR, SBP, stroke volume, and cardiac output [38]. Our study found that a combination of DEX-tiletamine-zolazepam did not significantly change cardiovascular parameters such as HR and SBP in both groups compared with baseline. A similar result was reported in a previous study in which dogs received a combination of dissociative drugs and DEX; a lower HR was not detected because sympathetic stimulation by dissociative drugs, either tiletamine or ketamine, alleviated the negative effects of DEX, resulting in a normal range of HR [39, 40]. Kilic and Erkut [41] studied the impacts of anesthesia in dogs using medetomidine, ketamine, and diazepam; HR, respiratory rate, and body temperature were investigated before anesthesia, at 15, 30, and 45 min during anesthesia, and at 24 h after anesthesia. The study showed a significant decrease in HR, respiratory rate, and body temperature after administering medetomidine, ketamine, and diazepam. In our study, however, IOP, HR, SBP, and SpO_2_ were measured in all dogs before premedication and at 5, 10, 15, and 20 min after premedication, immediately after tiletamine-zolazepam administration, after endotracheal intubation, and at 72 h post-operative. We conducted a prospective study based on Kilic and Erkut [41] and investigated the parameters during the anesthesia period.

The increase in IOP may result from one factor or the combination of induction drug and intubation [42, 43]. A previous study reported that IOP was significantly higher immediately after induction and after endotracheal intubation [44]. This study showed that IOP, HR, and SBP increased after tiletamine-zolazepam administration, but these values were not differ significantly from the baseline levels. The dissociative drug administration causes symptomatic stimulation, increasing HR [14], SBP [15], and IOP [18]. However, Jang *et al*. [16] reported the effects of different doses of tiletamine-zolazepam on IOP in healthy beagle dogs; the authors found that intravenous administration at 5, 10, and 20 mg/kg of tiletamine-zolazepam had no significant effect on IOP. Similarly, IOP did not significantly change in cats administered 10 mg/kg of tiletamine-zolazepam by intranasal and intramuscular routes [45]. Furthermore, premedication with DEX blunts the increase in IOP induced by dissociative drugs, laryngoscopy, and intubation [25].

Numerous previous studies have described an increase in IOP after laryngoscopy and endotracheal intubation above pre-insertion values in humans [46] due to symptomatic stimulation [47, 48], whereas receiving DEX premedication decreased the pressor response to laryngoscopy and intubation [25]. In our study, dogs were intubated after induction with tiletamine-zolazepam to protect the airway and avoid prolonged apnea, regurgitation, and pulmonary aspiration of the gastric content [49]. Thus, we could not separate the effect of the induction drug from that of endotracheal intubation on increased IOP, HR, and SBP. Nevertheless, in a study by Ismail *et al*. [46], IOP, HR, and SBP showed a lower change at 1 and 2 min after insertion of an endotracheal tube, without significant differences from baseline [50]. However, these parameters were not measured continuously in our study.

Siegenthaler *et al*. [51] described profound sedation in all dogs administered intravenously administered 10 μg/kg DEX. Moreover, DEX combined with an opioid [20, 52] or another anesthetic drug [35] resulted in greater sedation than DEX alone. In our study, DEX administration alone significantly increased the level of sedation compared with baseline (indicating moderate sedative score; 6–12). These drugs, combined with tiletamine-zolazepam, induced relatively heavy sedation (indicating heavy score sedation; 13+) in both groups and a SpO_2_ decrease in the groups receiving 10 μg/kg of DEX. However, SpO_2_ returned to baseline after endotracheal intubation. The previous studies have shown that tiletamine-zolazepam combined with an alpha-2 adrenergic agonist caused some degree of respiratory depression [53, 54], and endotracheal intubation plus supplemental oxygen must be received to prevent hypoxemia [53, 55]. Furthermore, post-operative measurements in our study showed the normal range of values in IOP, HR, SBP, SpO_2_, and sedation scale.

However, a limitation of our study is that IOP was not measured during the surgical procedure because these dogs were positioned in dorsal recumbency for ovariohysterectomy or castration.

## Conclusion

Injecting DEX at doses of 5 and 10 μg/kg produced sedation, and higher sedation was observed when combined with tiletamine-zolazepam. Moreover, intramuscular administration of DEX alone (10 μg/kg) induced a significant reduction in IOP and HR but without significantly influencing SBP. Intravascular tiletamine-zolazepam administration caused no changes in IOP, HR, and SBP compared with baseline. Therefore, DEX administered intramuscularly at a dose of 10 μg/kg is a suitable premedication for ocular examination or surgery to avoid undesirable IOP increase in dogs. Moreover, the results confirm that DEX combined with tiletamine-zolazepam is another option for ophthalmic surgery in dogs as it has no clinically significant effects on IOP or cardiovascular parameters.

## Authors’ Contributions

JS and PK: Conceptualization, design of the study, and methodology. JS, PK, and TS: Sample collection. JS and PK: Data analysis and writing-original draft, and manuscript review. JS, PK, and TS: Manuscript editing. All authors have read and approved the final manuscript.
